# Distributions of Polychlorinated Naphthalenes in Sediments of the Yangtze River, China

**DOI:** 10.3390/molecules26175298

**Published:** 2021-08-31

**Authors:** Zhitong Liu, Ke Xiao, Jingjing Wu, Tianqi Jia, Rongrong Lei, Wenbin Liu

**Affiliations:** 1 State Key Laboratory of Environmental Chemistry and Ecotoxicology, Research Center for Eco-Environmental Sciences, Chinese Academy of Sciences, Beijing 100085, China; ztliu@rcees.ac.cn (Z.L.); jingwoo@rcees.ac.cn (J.W.); tqijia@126.com (T.J.); leirongr@163.com (R.L.); liuwb@rcees.ac.cn (W.L.); 2 College of Resources and Environment, University of Chinese Academy of Sciences, Beijing 100049, China; 3 Hangzhou Institute for Advanced Study, University of Chinese Academy of Sciences, Hangzhou 310024, China

**Keywords:** PCNs, spatial distribution, congener, environmental risk, sediment

## Abstract

The pollution status of polychlorinated naphthalenes (PCNs) in the sediment of the Yangtze River Basin, Asia’s largest river basin, was estimated. The total concentrations of PCNs (mono- to octa-CNs) ranged from 0.103 to 1.631 ng/g. Mono-, di-, and tri-PCNs—consisting of CN-1, CN-5/7, and CN-24/14, respectively, as the main congeners—were the dominant homolog groups. Combustion indicators and principal component analysis showed that the emissions from halowax mixtures were the main contributor to PCNs in sediment, among most of the sampling sites. The mean total toxic equivalent (TEQ) was calculated to be 0.045 ± 0.077 pg TEQ/g, which indicates that the PCNs in sediments were of low toxicity to aquatic organisms. This work will expand the database on the distribution and characteristics of PCNs in the river sediment of China.

## 1. Introduction

Polychlorinated naphthalenes (PCNs) have been synthesized since the 1930s, with 75 congeners based on the number and position of the chlorine(s) in the naphthalene ring system, including 2 mono-chlorinated (CN-1–CN-2), 10 di-chlorinated (CN-3–CN-12), 14 tri-chlorinated (CN-13–CN-26), 22 tetra-chlorinated (CN-27–CN-48), 14 penta-chlorinated (CN-49–CN-62), 10 hexa-chlorinated (CN-64–CN-72), 2 hepta-chlorinated (CN-73–CN-74), and 1 octa-chlorinated (CN-75) [1]. Because of the properties of low water solubility, low vapor pressure, and resistance to degradation, PCNs were widely used in various industries including cable insulation, wood preservation, graphite electrode lubrication, masking compounds for electroplating, dye manufacturing, capacitors, and refracting index testing oils [1,2]. In parallel, an increasing number of toxicological studies have demonstrated that PCNs exhibited the potential risk to a variety of organisms, including dioxin-like toxic effects on mammals [3,4,5]. PCNs have been listed in Annexes A and C of the Stockholm Convention on Persistent Organic Pollutants since May 2015, owing to their potential toxicity, persistence, bioaccumulation, and long-range transport [6,7]. Although, the production of PCNs was banned in the 1980s [2], unfortunately, products containing PCNs have been unlawfully used so far [8]. There are mainly four pathways of PCNs discharging into the environment, including historical use, historical and present use of polychlorinated biphenyl (PCBs), thermal and other processes, and landfills [9]. Moreover, PCNs may also be formed unintentionally and emitted into the environment via thermal processes involved in nonferrous metallurgical facilities, metal refineries, waste incineration, and cooking industries [9,10,11]. The possible sources of PCNs released into the environment could be identified by analyzing homolog profiles and ratios of several PCN congeners used as indicators of particular emission activities [11].

PCNs have been found in various media such as soil, sediment, water, air, and biota, even in human breastmilk [12,13]. Sediment, as one of the most important pollutant sinks among the environmental media, can hold the contemporary PCNs from aquatic and terrestrial sources and release them to environment again. Thus, it is essential to monitor PCNs in sediment as secondary pollution sources in order to examine their ecological risk. In fact, detectable PCNs have been reported in numerous sediment samples collected in rivers, lakes, and coastal waters of China [7,14,15,16,17,18], even though PCNs have not been intentionally produced in China. For example, in Zhang’s study, the PCN concentration was up to 4610 ng/g, highest among all the sediment samples of China reported [16]. Moreover, these pollutants tend to long-range transport in the atmosphere and finally deposit to sediments. Therefore, it is necessary to conduct comprehensive research on the whole valley for accurate assessment of the extent of pollute.

The Yangtze River, Asia’s longest river and the third longest river in the world, serves as an important resource for drinking water, aquaculture and industrial use. With a rapid increase in population and economic development around the river, there are numerous inputs from industrial wastewater, municipal sewage, atmospheric deposition, as well as agricultural soils containing fertilizers, pesticides, herbicides, and heavy metals [19]. Preserving the river’s water quality is critical for sustainable development as well as the health and survival of residents. In this study, sediment samples of the Yangtze River Basin were collected from the cradle to the estuary, including the reservoirs, industrial zones and residential areas. The samples spanned more than 6000 km across six provinces at different levels of economic development. Our aims were to provide fundamental data for quantifying PCN concentrations in the sediments, establishing their distributions and characteristics, and evaluating their potential ecological risks.

## 2. Results and Discussion

### 2.1. Spatial Distributions of PCNs in Sediments

**Figure 1:**
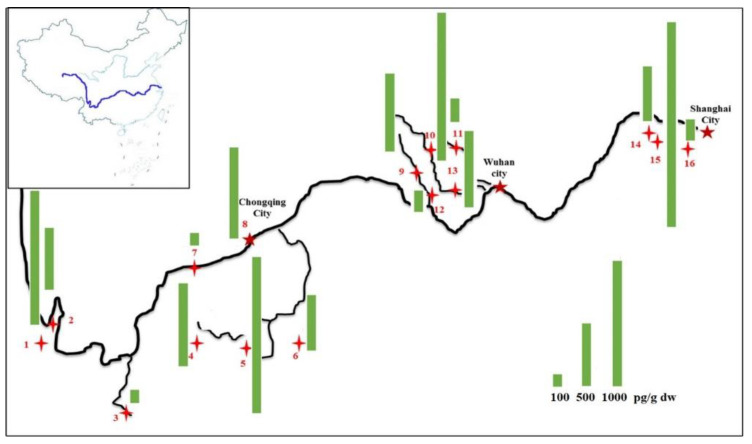
The PCN concentrations of the sampling sites from the Yangtze River Basin.

The PCN concentrations of the sampling sites from the Yangtze River basin are shown in Figure 1.

The PCN concentrations in the sediment samples ranged from 0.103 to 1.631 ng g^−1^. The lowest concentration of PCNs was found at S7 located in Minjiang River which was served as a water reservoir. The highest concentration of PCNs was found at S15, collected from Xijiu River in Yixing City, a residential urban area. In the upstream zone (samples S1–S3), the relatively higher PCN concentration was found at S1 located near a famous tourist resort. Among the upper- and middle-stream zone samples, the PCN concentrations of S8 and S5 were relatively high. This was mainly due to the fact that S8 was in a large city, and S5 was close to an industrial development zone. Within the sample from the middle and lower zones, the concentration of PCNs was relatively high at S10, which was near the outlet of a sewage treatment plant. As mentioned in the previous study [20], the input of wastewater and sewage sludge from urban treatment plants might be the most significant source of PCNs in the sediment. In the downstream zones, the highest concentration of PCNs was at S15, which was the inlet of Taihu Lake. The lake has become a pollutant sink with rapid urbanization and subsequently increasing amount of agricultural and industrial waste [16]. In our study, S3, S7, S12 and S16 could all be treated as background with lower concentrations of PCNs (0.1–0.2 ng g^−1^). Unlike results reported from Li et al. [7], the trend in PCN concentrations at various spots above did not increase from cradle to the estuary. Therefore, PCN concentrations in sediments were more closely related to local human activities and industrial sources rather than geographically locations.

### 2.2. Comparison with Reported PCN Levels of Other Studies

PCN levels in this study were compared with those reported from other parts of the world (Table 1). Sample S16 in our study in Suzhou City was near the sampling point from the study of Zhang et al. [16], who reported that the PCN concentrations in the sediment was up to 4610 ng/g·dw in development zone of major industrial plants located in downtown Suzhou City. In contrast, our measured value (0.17 ng/g·dw) was five orders of magnitude lower than the above mentioned, while the sampling sites were at such a close distance about 25 miles away. It might because S16 was collected from a drinking water source in the suburb of Suzhou City. This result confirmed that local human activities and industrial sources strongly influence the PCN concentrations.

Compared with other locations, the PCN concentrations in our study were the same order of magnitude as in the Venice Lagoon in Venice (0.03–1.15 ng/g·dw), the Gulf of Bothnia in Sweden (0.088–1.9 ng/g·dw), the Liaojie River in Taiwan (0.029–0.987 ng/g·dw), the Tokyo Bay in Japan (1.81 ng/g·dw), the Qingdao Coastal Sea in China (1.1–1.2 ng/g·dw), and Laizhou Bay in China (0.12–5.10 ng/g·dw) [8,18,21,22,23,24]. Results herein were also lower than those in the Liao River basin in China (0.33–12.5 ng/g·dw), the Yellow River in China (0.18–130 ng/g·dw), and the Danube catchment in Czech Republic (0.05–29.2 ng/g·dw) [7,25,26]. They were significantly lower than that in the Bitterfeld and coastal Georgia, with chloralkali industries nearby as a source of PCNs [27,28]. The PCN concentrations in the sediments of the Yangtze River Basin were relatively lower, for the reason that some samples were collected from the water source protection area or the rural areas.

**Table 1:** Polychlorinated naphthalene levels reported in other parts of the world.

Countries/River Side	Sediment Concentration (ng/g·dw)	TEQ * (pg/g)	Reference
Georgia coastal	23400	3.71 × 10^6^	[27]
Bitterfeld, Germany	2540	/	[28]
Swedish Lake	0.1–7.6	/	[29]
Gdansk Basin, Baltic Sea	6.7	/	[22]
Venice Lagoon, Venice	0.03–1.15	0.01–0.22	[21]
Tokyo Bay, Japan	1.81	/	[8]
Gulf of Bothnia, Sweden	0.088–1.9	/	[30]
Baltic Sea	0.32–1.9	0.19–0.23	[31]
Qingdao Coastal Sea, China	0.212–1.21	0.04–0.38	[24]
Barcelona, Spain	0.17–6.56	/	[32]
Lake Ontario	21–38	17 ± 4	[33]
Laizhou Bay, China	0.12–5.10	/	[18]
Jiaozhou Bay, China	0.0039–0.00564	<0.1	[14]
River Chenab, Pakistan	8.94–414	0.1–57	[34]
Danube Catchment, Czech Republic	0.05–29.2	0.02–17	[26]
Yangtze River Delta, East China	0.6–4600	0.0014–2160	[16]
Liao River, China	0.33–12.5	/	[7]
Yellow River, China	0.618–130	/	[24]
East China Sea	0.002–261.71	0–212	[35]
Liaojie River, Taiwan	0.029–0.987	3.4 × 10^−3^–0.71	[23]
Yangtze River, China	0.103–1.631	0.010–0.304	This study

* TEQ: total toxic equivalent.

### 2.3. PCN Homolog Profiles

**Figure 2:**
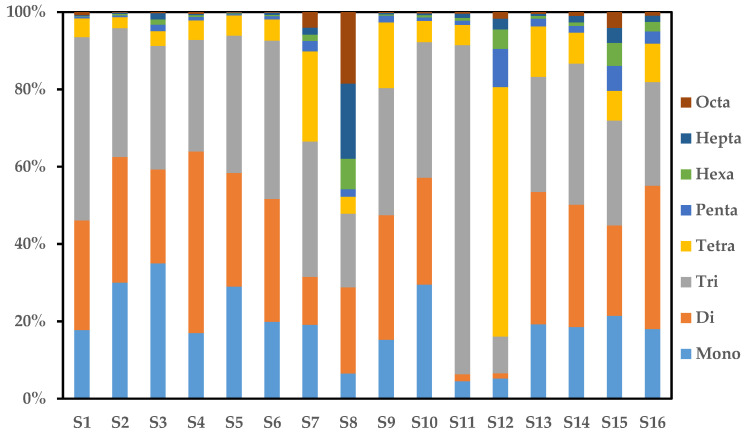
Homolog profiles of polychlorinated naphthalene in the sediment samples from the Yangtze River basin.

PCN homolog profiles for the environmental samples may be of great help to qualitatively identify the sources. Figure 2 shows the PCN homolog profiles of the sediment samples from the Yangtze River Basin. All of 75 PCN congeners were detected by high-resolution gas chromatography/high-resolution mass spectrometry. In other studies, PCNs homologs were usually reported the results of tri- to octa-CNs, with the dominant homologs of tri-and tetra-CNs, such as the Liaohe River basin of China, Laizhou Bay of China, and the Yellow River of China [7,18,25]. By contrast, in our study, mono-, di-, and tri-CNs were the dominant homolog groups in most of the samples, with the CN-1, CN-5/7 (i.e., complex of CN-5 and CN-7), and CN-24/14 being dominant congeners. Moreover, the proportion of PCN homolog groups decreased with an increasing number of chlorine atoms. Experimental results showed that S8 dominated by hepta-and octa-CN homologs, while S11 and S12 were dominated by tri-and tetra-CN homologs. S8 was collected from the stem of the Yangtze River, which was near the developed cities Chongqing City. This sampling site was surrounded by large commercial streets, busy transport hubs, and a garment industry. S11 was collected in Yunshui River near a fertilizer plant. S12 was collected from Changhu Lake surrounded by the woodland near Jingmen City. Although there was a sewage treatment plants that may discharge waste into the lake, the Jingmen City gathered a few industries including battery factories, petroleum machinery factories, etc. Therefore, we speculated that the significant difference in the distribution of PCN congeners in S11 and S12 samples may be due to the transport and deposition of surrounding industrial sources. The local hydrologic monitoring stations reported that the flow rate in S12 was much lower than in S11. Therefore, the different distribution of the PCN congeners in these three sites may result from human activities, industry discharges as well as the hydrological characteristics, deserving further investigations.

### 2.4. Source Identification

**Figure 3:**
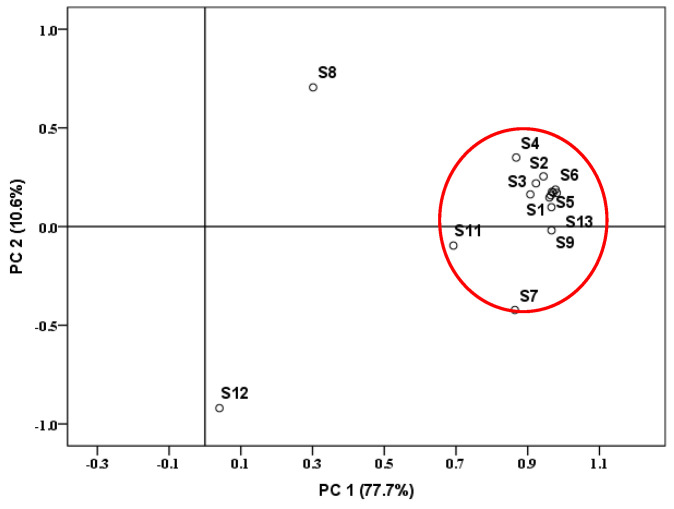
PCA score plot of the polychlorinated naphthalene profile of sediment samples.

To further identify the potential sources of PCNs in the samples, we conducted principal component analysis (PCA) on PCN homologs in the sediment samples of the Yangtze River. Figure 3 shows the results of the PCA score plot of PCN among different samples. The extraction variance contributions of PC1 and PC2 accounted for 77.7% and 10.6% of the total variance, respectively, with a cumulative value of 88.3%. Except for two samples (S8 and S12), the homolog of PCNs in other samples was relatively consistent (marked in the red circle), indicating that they may be from the same types of emission sources.

Several congeners—such as CN-17/25, -36/45, -39, -35, -52/60, -50, -51, -53, and -66/67—have been identified as combustion indicators (PCN_com_) [3,9,15,36]. The ratio of ∑PCN_com_ to ∑PCNs was usually calculated to estimate primary sources [36]. Values of ∑PCN_com_/∑PCNs < 0.11 suggested emission from the halowax mixture, values of ∑PCN_com_/∑PCNs > 0.5 indicated combustion-related source emissions, and 0.11 < ∑PCN_com_/∑PCNs < 0.5 was assumed for the indication of emissions from combustion sources and halowax products [15]. In this study, the values of ∑PCN_com_/∑PCNs were all lower than 0.11, except for S12 (0.19). Thus, it was speculated that the dominant sources of the PCNs were mainly from the emission of the halowax mixture. Besides, the concentration of PCNs in S12 were also affected by combustion-related source emissions. Even though PCN mixtures were never historically produced and are not currently in commercial use in China, the historic usage of halowax mixture such as paintings and rubber materials could still be a big contributor to PCNs of the sediment in the Yangtze River Basin [18].

### 2.5. Ecological Risks of PCNs in Sediments

**Figure 4:**
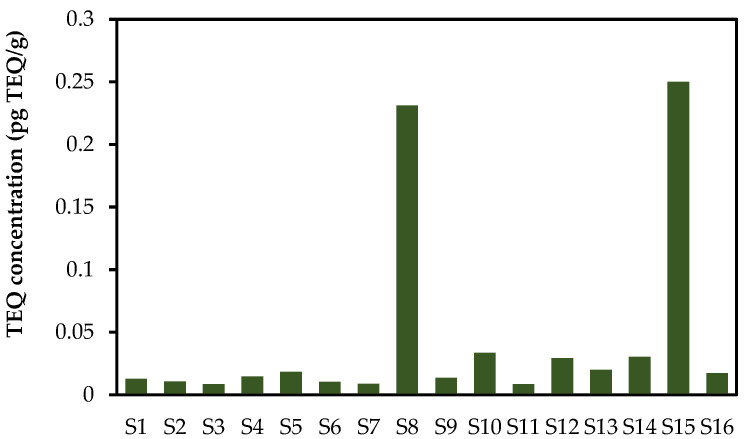
TEQ concentrations of the sediments in the Yangtze River Basin.

Some PCN congeners have toxic effects similar to those of 2,3,7,8-tetrachlorobenzo-*p*-dioxin (TCDD) in terms of their biological actions in animals [37]. Based on the relative potency factors [3], the calculated PCN-corresponding total toxic equivalent (TEQ) values in sediments ranged from 0.009 to 0.250 pg TEQ/g, as presented in Figure 4. Herein, the TEQ values were significantly lower than those in the interim sediment and quality guidelines in Canada and the USA (0.85 pg TEQ/g and 2.5 pg TEQ/g) [17], meaning that PCNs in the sediments expressed very low toxicity to aquatic life. Highest TEQ values were found at S8 and S15, which were nearly ten times higher than those of the other samples. CN-73 concentrations contributed the most to the TEQs, with 54.8 and 37.4 pg/g·dw, respectively.

Compared with previous studies, the TEQ values in this study (Table 1) were several orders of magnitude lower than those reported for the Danube catchment (17 pg TEQ/g), Lake Ontario (17 ± 4 pg TEQ/g), the River Chenab in Pakistan (57.1 pg TEQ/g), and the Yangtze River Delta in China (2160 pg TEQ/g) [16,26,33,34]. Rather, our TEQ values were similar to those of the Venice Lagoon of Venice, the Qingdao Coastal Sea of China, Jiaozhou Bay of China, and the Liaojie River of Taiwan [14,21,23,24].

## 3. Materials and Methods

### 3.1. Sampling

To investigate the distribution of PCNs in the sediments along the Yangtze River Basin, a series of sediments was collected from 16 typical sites, including rural, urban and industrial areas based on their surrounding environment and levels of economic development nearly (Table 2).

**Figure 5:**
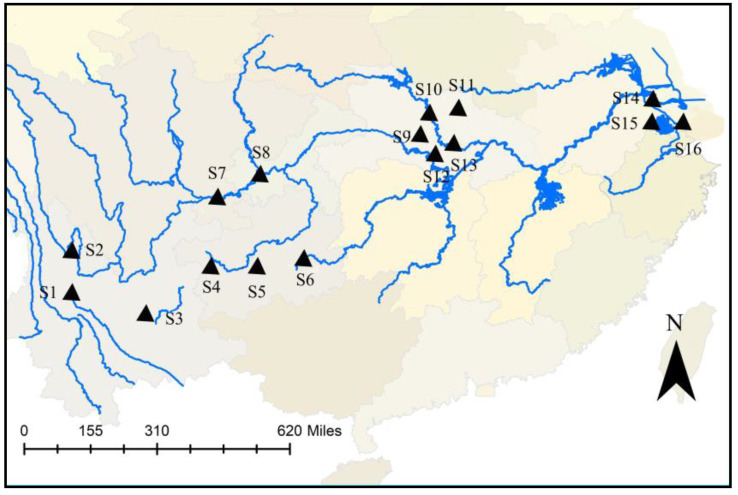
Yangtze River sampling sites in this study.

This picture just showed Level I and II tributaries. With the direction of the river, the samples could also be divided in four major zones: the upstream (Samples S1–S3), the upper and middle-stream (Samples S4–S8), the middle and lower (Samples S9–S13), and the downstream (Samples S14–S16) (Figure 5). According to the sampling method reported [21], the surface sediments (from the top 0–20 cm) were collected using a grab sampler, transported to the laboratory, and stored at −20 °C until analysis.

### 3.2. Sample Preparation and Analysis

The PCNs were analyzed according to our previously described method [20]. Briefly, the sediments were freeze-dried for approximately 48 h and passed through a 100-mesh (150 μm) stainless steel sieve. Approximately 10 g of sediments was weighed and spiked with a mixture of ^13^C_10_-labeled PCN internal standards (ECN-5102, tetra-octa PCNs Mixture composed of ^13^C_10_-CN-27, -42, -52, -67, -73, and -75; Cambridge Isotope Laboratories, Andover, MA, USA) and then mixed with 40 g (dry weight) of diatomaceous earth, and extracted by accelerated solvent extraction (ASE 350; Thermo-Fisher Scientific, Waltham, MA, USA) at 120 °C with a mixture extraction solvent of hexane and dichloromethane (1:1, *v*:*v*). The extraction solvents were first cleaned using acid silica column, followed by multilayer silica gel column and then basic alumina column. The elution fraction was then concentrated to 20 µL by rotatory evaporation and a gentle nitrogen gas stream. Finally, ^13^C_10_-labeled PCN (ECN-5260: ^13^C_10_-CN-64, Cambridge Isotope Laboratories, United States) was spiked for the calculation of recoveries before analysis.

PCNs were analyzed by high resolution gas chromatography coupled with a high-resolution mass spectrometer (Thermo Fisher Scientific, Waltham, MA, USA). A DB-5 fused silica capillary column (60 m × 0.25 mm × 0.25 μm; Agilent Technologies, Santa Clara, CA, USA) was used for the separation of PCN congeners. The injection volume was 1 μL, the flow rate of helium as carrier gas was 1 mL min^−1^, and GC inlet temperature was set at 270 °C. The temperature program was initiated at 80 °C (for 2 min) and increased to 180 °C at 20 °C min^−1^ (hold for 1 min), 280 °C at 2.5 °C min^−1^ (for 2 min), and 300 °C at 10 °C min^−1^ (for 5 min). The HRMS was tuned and operated at approximately 10,000 resolutions with 45 eV EI energy.

### 3.3. Quality Assurance and Quality Control (QA/QC)

A procedural blank sample was evaluated to assess the possible contamination and instrumental stability. Only a small amount of monochlorinated polychlorinated naphthalenes was detected in the blank samples and was 10% lower than the concentrations in sediment samples. The PCN concentrations in the samples were, thus, not corrected using the values from the blanks. The recoveries of the ^13^C_10_-labeled congeners ranged from 62% to 98%. The instrumental detection limits were assimilated when the signal-to-noise ratio was three times. PCNs were quantified using a relative response factor of the labeled congener at the same level of chlorination and a similar retention time.

## 4. Conclusions

We evaluated the distribution, composition, and ecological risks of PCNs by analyzing 16 sediment samples of the Yangtze River basin from the cradle to the estuary. Their concentrations and TEQs were less than 2 ng g^−1^ and 0.3 pg TEQ/g, respectively. These levels were lower than those of most of the previous reports, demonstrating that there was nearly no alarm to aquatic life with toxic aspects. In our study, the relatively higher PCN concentrations and TEQs were generally related with frequent human activities and nearby industrial sources. Further research is needed, however, to elucidate the relationship between concentrations of PCN congeners and human activities, providing new insights into understanding the environmental and health risk of exposure to PCN at low level.

## Figures and Tables

**Table 2:** Geographical information and site types for the 16 sampling sites on the Yangtze River Basin.

Number	Sites Name	Latitude	Longitude	Type of Sample Site
S1	Erhai	25°37′	100°15′	city
S2	Sansu River	26°59′	100°13′	rural
S3	Dianchi	24°54′	102°43′	rural
S4	Xiangshui River	26°34′	104°56′	city
S5	Hongfenghu Reservoir	26°34′	106°26′	industrial areas
S6	Qingshui River	27°31′	109°35′	city
S7	Min River	28°45′	104°38′	city
S8	Stem Stream of Yangtze River	29°32′	106°34′	developed city
S9	Zhanghe Reservoir	30°58′	112°04′	rural
S10	Man River	31°35′	112°16′	city
S11	Runshui River	31°43′	113°20′	city
S12	Chang Lake	30°23′	112°30′	woodland
S13	Hanbei River	30°40′	113°08′	rural
S14	Tao Lake	31°61′	119°55′	rural
S15	Xi Jiu River	31°21′	119°48′	city
S16	Cheng Lake	31°18′	120°51′	rural

## Data Availability

Not applicable.

## Sample Availability

Samples of the compounds are not available from the authors.
